# A systematic pan-cancer analysis reveals the clinical prognosis and immunotherapy value of C-X3-C motif ligand 1 (CX3CL1)

**DOI:** 10.3389/fgene.2023.1183795

**Published:** 2023-04-20

**Authors:** Yidi Sun

**Affiliations:** School of Biomedical Engineering, Hainan University, Haikou, Hainan, China

**Keywords:** chemokines, CX3CL1, pan-cancer, prognosis, immune infiltration

## Abstract

It is now widely known that C-X3-C motif ligand 1 (CX3CL1) plays an essential part in the process of regulating pro-inflammatory cells migration across a wide range of inflammatory disorders, including a number of malignancies. However, there has been no comprehensive study on the correlation between CX3CL1 and cancers on the basis of clinical features. In order to investigate the potential function of CX3CL1 in the clinical prognosis and immunotherapy, I evaluated the expression of CX3CL1 in numerous cancer types, methylation levels and genetic alterations. I found CX3CL1 was differentially expressed in numerous cancer types, which indicated CX3CL1 may plays a potential role in tumor progression. Furthermore, CX3CL1 was variably expressed in methylation levels and gene alterations in most cancers according to The Cancer Genome Atlas (TCGA). CX3CL1 was robustly associated with clinical characteristics and pathological stages, suggesting that it was related to the degree of tumor malignancy and the physical function of patients. As determined by the Kaplan-Meier method of estimating survival, high CX3CL1 expression was associated with either favorable or unfavorable outcomes depending on the different types of cancer. It suggests the correlation between CX3CL1 and tumor prognosis. Significant positive correlations of CX3CL1 expression with CD4^+^ T cells, M1 macrophage cells and activated mast cells have been established in the majority of TCGA malignancies. Which indicates CX3CL1 plays an important role in tumor immune microenvironment. Gene Ontology (GO) terms and the Kyoto Encyclopedia of Genes and Genomes (KEGG) pathway enrichment analysis suggested that the chemokine signaling pathway may shed light on the pathway for CX3CL1 to exert function. In a conclusion, our study comprehensively summarizes the potential role of CX3CL1 in clinical prognosis and immunotherapy, suggesting that CX3CL1 may represent a promising pharmacological treatment target of tumors.

## 1 Introduction

Chemokines typically serve to attract and facilitate the movement of leukocytes to inflammatory sites in the body (Zlotnik et al., 2006; Nagarsheth et al., 2017; Pawluczuk et al., 2020). Over fifty chemokines have been identified in humans, and chemokine signaling is mediated and transmitted by chemokines and their respective receptors (Groblewska et al., 2020; Huynh et al., 2020). T lymphocytes, macrophages, neutrophils, epithelial cells and tumor cells are all possible sources of chemokines, while tumor-associated macrophages (TAMs) and tumor cells show preferential expression of chemokine receptors, indicating their potential roles in carcinogenesis (Subileau et al., 2009; Hughes and Nibbs, 2018; Sharma et al., 2018; Blank et al., 2021; Tarek et al., 2021; Ye et al., 2021). Chemokines have been demonstrated to mediate inflammation and regulate cells adhesion, proliferation, and migration (Zhang et al., 2021a; Qin et al., 2022).

Due to the location of two cysteines at the N-terminus, chemokines can be classified into a number of distinct subfamilies (Hughes and Nibbs, 2018; Ye et al., 2021). CX3CL1 (C-X3-C motif ligand 1) is the sole member of the CX3C chemokine subfamily (Qin et al., 2022). CX3CL1 is a chemokine with multiple functions that is encoded on human chromosome 16q21 by the CX3CL1 gene (Chapman et al., 2000). According to reports, macrophages, neurons, epithelial and dendritic cells (DCs), and natural killer cells (NKs) constitutively express CX3CL1 (Patel et al., 2011). CX3CR1 (CX3C chemokine receptor 1), in contrast, is widely expressed by cytokine-producing cells, including Th cells, TAMs, cytotoxic T cells, monocytes, DCs, and NKs (Liu et al., 2005; Ao et al., 2022a). Therefore, the CX3CL1-CX3CR1 axis can facilitate the progression of various cancers by regulating the recruitment of multiple immune cells. CX3CL1 normally exerts its function through two distinct isoforms: membrane-anchored form and soluble form. The membrane-anchored form plays a regulatory role via constitutive internalization by maintaining dynamic equilibrium between the plasma membrane and the intracellular endocytic compartment (Chapman et al., 2000; Liu et al., 2005; Patel et al., 2011; Gai et al., 2021; Nannini et al., 2021; Ao et al., 2022a). Additionally, the soluble form act as an essential chemoattractant for DCs, NK cells, and T cells (Dichmann et al., 2001; Umehara et al., 2001). It has been demonstrated that CX3CL1 has a tumor suppressing effect (Guo et al., 2002; Guo et al., 2003). On the other hand, CX3CL1 promotes cancer cells proliferation and migration, and contributes to angiogenesis, which were significantly inhibited by the administration of CX3CL1-neutralizing antibodies (Liang et al., 2018; Liu et al., 2018; Liu et al., 2019a; Yu et al., 2022a; Ao et al., 2022b). Previous research has demonstrated that CX3CL1 plays a crucial function in numerous types of cancers, including ovarian carcinoma (Singh et al., 2019), B-cells lymphoma (Corcione et al., 2010), neuroblastoma (Nevo et al., 2009), breast cancer (Onitilo et al., 2009), gastric cancer (Lv et al., 2014), pancreatic cancer (Xu et al., 2012; Ran et al., 2020), prostate cancer (Tang et al., 2016), colorectal cancer (Zheng et al., 2013), lung cancer (Schmall et al., 2015), and hepatocellular cancer (Huang and Geng, 2010). However, no comprehensive investigation of CX3CL1 across all The Cancer Genome Atlas (TCGA) tumor types has been performed.

To date, research on CX3CL1 in cancer has been limited to a single type of cancer. Therefore, a thorough examination of CX3CL1 in cancer is required to determine its association with clinical phenotypic characteristics, tumor prognosis, and tumor immune infiltration. The genome-wide pan-cancer analysis is advantageous for elucidating the correlation between the expression of CX3CL1 and carcinogenesis, which in turn informs the development of more effective surveillance, diagnosis, and treatment strategies. Consequently, the present study utilized the TCGA database to analyze CX3CL1 in all TCGA cancers, providing a comprehensive picture of this important chemokine, taking into account multiple aspects, including gene expression, predictive value, genetic mutation, tumor immune infiltration, and enrichment analyses.

CX3CL1 showed significant differential expression in approximately 58% of TCGA cancer types, according to the results. Approximately 32% of the cancer types with significantly differential expression were adenocarcinomas, suggesting a specific role for CX3CL1 in adenocarcinomas. Approximately 45% of TCGA cancer types were associated with CX3CL1 methylation, and 60% of these cancer types displayed significant differential CX3CL1 expression. Seventy percent of TCGA cancer types had CX3CL1 mutations, of which more than half were significantly associated with CX3CL1 expression. The three main clinical outcome endpoints for survival analysis in this study were overall survival (OS), disease specific survival (DSS), and progression free interval (PFI). High CX3CL1 expression was associated with favorable prognosis in CESC and KICH, suggesting that CX3CL1 may be a potential biomarker for prediction the prognosis of these two cancer types. CX3CL1 expression was positively correlated with the infiltration levels of CD4^+^ T cells, M1 macrophage cells, and activated mast cells in various cancers. In COAD and HNSC, the expression of CX3CL1 was positively correlated with the infiltration levels of the three aforementioned immune cells, indicating that CX3CL1 plays a more significant role in the immune microenvironment of these two cancers. These findings pave the way for a greater comprehension of the role of CX3CL1 in tumor prognosis and immune therapy.

## 2 Materials and methods

### 2.1 TIMER2.0 database

The TIMER2 database is an all-encompassing database for studying immune infiltrates in cancers (Li et al., 2020). The website provides immune infiltration abundances calculated by multiple algorithms. Several modules of TIMER2 database were involved in this work. Differential expression analysis was conducted using the “Gene_DE module.” Meanwhile, the “Immunological-Gene module” investigated CX3CL1 expression levels in relation to infiltrating immune cells. Of note, CD4^+^ T cells, macrophage, and mast cells were chosen for further study. Furthermore, the “Gene_Corr” unit was employed to reveal a relationship between CX3CL1 and particularly chosen genes present in all TCGA cancers.

### 2.2 GEPIA2 database

The GEPIA2 database is a promising tool offering a number of configurable features for users to investigate (Tang et al., 2019). Participating were normal samples collected as part of the GTEx (Genotype-Tissue Expression) project. Utilizing the “Expression Analysis-Box Plot” unit, a comparison of the CX3CL1 expression for a variety of cancer types with or without normal tissues was performed. In addition, using the “Expression Analysis-Pathological Stage Plot” unit, a pathological stage analysis of CX3CL1 expression was performed. Additionally, the “Expression Analysis-Similar Genes Detection” unit was utilized to identify the genes associated with CX3CL1.

### 2.3 UALCAN database

The UALCAN database is a comprehensive and interactive tool for OMICS data analysis on cancer (Chandrashekar et al., 2022). In the context of epigenetic regulation, UALCAN investigates the effect of promoter methylation on gene expression in order to learn more about the targets of interest and collect pertinent data. In our study, I measured and compared the CX3CL1 promoter methylation levels in various cancers.

### 2.4 cBioPortal database

Researchers can convert multidimensional cancer genomics datasets into biological insight and clinical applications using the cBioPortal database (Cerami et al., 2012). For the purposes of this research, the “TCGA Pan Cancer Atlas Studies-Cancer Types Summary” unit was consulted in order to acquire information regarding mutation types, copy number alterations (CNA), and precise alteration frequencies for CX3CL1 in pan-cancer.

### 2.5 STRING database

The STRING database collects, scores, and integrates information on protein-protein interactions (PPI) (Szklarczyk et al., 2019; Chen et al., 2020; Dholaniya and Rizvi, 2021; Cui et al., 2022a; Chen et al., 2023). The purpose of the website is to develop a comprehensive and unbiased global network that incorporates both physical and functional interactions. A total of 20 genes that interact with CX3CL1 were then uncovered in this work.

### 2.6 Functional annotations

In order to carry out enrichment analyses, such as Gene Ontology (GO) and Kyoto Encyclopedia of Genes and Genomes (KEGG) pathway analyses, the “clusterProfiler” and “enrichplot” R packages were utilized (Ashburner et al., 2000; Kanehisa and Goto, 2000; Yu et al., 2021). Kyoto Encyclopedia of Genes and Genomes pathway analysis was using “enrichKEGG” function. Adjusted ‘*p* = 0.05’ and ‘Q = 0.5’ were selected as the cut-off value. Gene ontology pathway analyses was using “enrichGO” function. Adjusted ‘*p* = 1’ and ‘Q = 1’ were selected as the cut-off value. Two function “barplot” and “dotplot” were used to construct plot.

### 2.7 Statistical analysis

Receiver operating characteristic curves (ROC curves) were used to examine the predictability of CX3CL1 between normal and tumor tissues (Yu et al., 2022b; Mostafa et al., 2022; Xiang et al., 2022). The R packages “randomForestSRC” and “randomSurvivalForest” were used to compute the area under the curve (AUC) to access the model’s accuracy. The performance of the model improved as the AUC got closer to 1 (AUC >0.8). The R packages “survival” and “survminer,” along with the Kaplan-Meier method, were employed to create survival curves utilizing clinical data as the basis. Overall survival (OS) was utilized to assess the patients’ outcomes. Disease specific survival (DSS) refers to the time period during which individuals die from a particular disease. The progression free interval (PFI) is the time during which a patient’s cancer does not worsen. I used univariate survival analysis to obtain the log-rank *p*-value, and I considered the *p*-value to be statistically significant if it was 0.05 or lower.

## 3 Results

### 3.1 CX3CL1 expression profiles in pan-cancer

**FIGURE 1:**
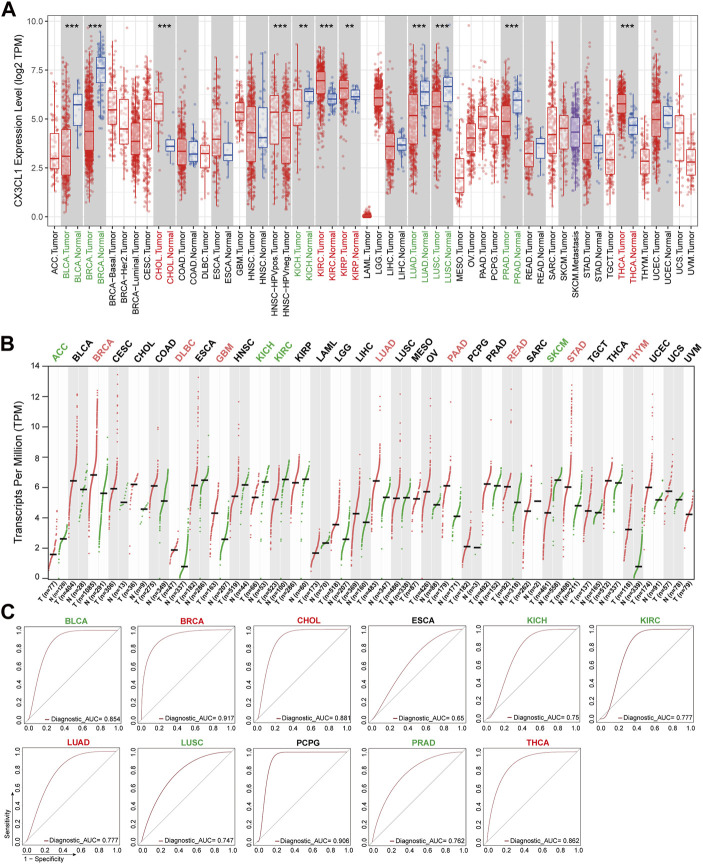
Aberrant expression of CX3CL1 across TCGA cancer types. **(A)** The TIMER2 database showed differential expression of CX3CL1 between various tumor tissues (red) and normal tissues (blue) in adrenocortical carcinoma (ACC), bladder urothelial carcinoma (BLCA), breast invasive carcinoma (BRCA), cervical squamous cell carcinoma and endocervical adenocarcinoma (CESC), cholangiocarcinoma (CHOL), colon adenocarcinoma (COAD), lymphoid neoplasm diffuse large B-cell lymphoma (DLBC), esophageal carcinoma (ESCA), glioblastoma multiforme (GBM), head and neck squamous cell carcinoma (HNSC), kidney chromophobe (KICH), kidney renal clear cell carcinoma (KIRC), kidney renal papillary cell carcinoma (KIRP), acute myeloid leukemia (LAML), brain lower grade glioma (LGG), liver hepatocellular carcinoma (LIHC), lung adenocarcinoma (LUAD), lung squamous cell carcinoma (LUSC), mesothelioma (MESO), ovarian serous cystadenocarcinoma (OV), pancreatic adenocarcinoma (PAAD), pheochromocytoma and paraganglioma (PCPG), prostate adenocarcinoma (PRAD), rectum adenocarcinoma (READ), sarcoma (SARC), skin cutaneous melanoma (SKCM), stomach adenocarcinoma (STAD), testicular germ cell tumors (TGCT), thyroid carcinoma (THCA), thymoma (THYM), uterine corpus endometrial carcinoma (UCEC), uterine carcinosarcoma (UCS), uveal melanoma (UVM). **p < 0.01; ***p < 0.001. **(B)** The GEPIA2 database showed differential expression of CX3CL1 in various tumor tissues (red) vs. normal tissues (green) based on The Cancer Genome Atlas (TCGA) and Genotype-Tissue Expression (GTEx) data. **(C)** Receiver operating characteristic (ROC) curves of different cancer types.

To investigate the potential role of CX3CL1 in the development of cancers, I analyzed the mRNA levels of CX3CL1 in a number of TCGA cancers included in the TIMER2 database. CX3CL1 expression was significantly elevated in cholangiocarcinoma (CHOL), kidney renal clear cell carcinoma (KIRC), kidney renal papillary cell carcinoma (KIRP), and thyroid carcinoma (THCA) tumor tissues compared to normal tissues (Figure 1A). Notably, HPV-positive head and neck squamous cell carcinoma (HNSC) tumor tissues expressed CX3CL1 significantly more than HPV-negative tumor tissues. Intriguingly, tumor tissues of bladder urothelial carcinoma (BLCA), breast invasive carcinoma (BRCA), kidney chromophobe (KICH), lung adenocarcinoma (LUAD), lung squamous cell carcinoma (LUSC), and prostate adenocarcinoma (PRAD) express CX3CL1 at significantly lower levels than normal tissues.

Due to the scarcity or low number of normal tissues of specific cancer types in the TIMER2 database, the basic expression level of CX3CL1 was also evaluated using the GTEx data from the GEPIA2 database. The tumor tissues of BRCA, lymphoid neoplasm diffuse large B-cell lymphoma (DLBC), glioblastoma multiforme (GBM), LUAD, pancreatic adenocarcinoma (PAAD), rectum adenocarcinoma (READ), stomach adenocarcinoma (STAD), and thymoma (THYM) contained significantly elevated levels of CX3CL1 (Figure 1B). CX3CL1 was less expressed in the tumor tissues of adrenocortical carcinoma (ACC), KICH, KIRC, and skin cutaneous melanoma (SKCM). In the remaining cancer types, there was no discernible difference in CX3CL1 expression between normal and malignant tissues, regardless of the inclusion or exclusion of the GTEx dataset.

CX3CL1 had significant differential expression in 19 out of 33 TCGA cancer types, including ACC, BLCA, BRCA, CHOL, DLBC, HNSC (HPV pos./neg.), GBM, KICH, KIRC, KIRP, LUAD, LUSC, PAAD, PRAD, READ, SKCM, STAD, THCA, and THYM, according to an analysis of two databases. BRCA, KICH, KIRC, and LUAD were the tumor types with significant differential expression of CX3CL1 in both databases. Given that the GEPIA2 database contains a larger sample size of normal controls, the GEPIA2 database’s results are regarded as more reliable.

Among the 19 cancer types with significant differential expression, those with higher CX3CL1 expression in tumor tissues than in normal tissues were categorized as upregulated, while those with lower expression were categorized as downregulated. BRCA, CHOL, DLBC, GBM, KIRP, LUAD, PAAD, READ, STAD, THCA, and THYM were among the upregulated group, while ACC, BLCA, KICH, KIRC, LUSC, PRAD, and SKCM were among the downregulated group. Other cancer types lacking a significant differential expression were deemed unrelated group.

In addition, the ROC curves demonstrated that CX3CL1 expression could differentiate between normal and tumor tissues (Figure 1C). The AUC values for BLCA, BRCA, CHOL, PCPG, and THCA were greater than 0.85, whereas those for KIRC, LUAD, and PRAD were greater than 0.75, indicating that these biomarkers have excellent predictive values for separating tumor tissues from adjacent normal tissues. These findings indicate that CX3CL1 is highly expressed in a variety of cancer types and is capable of identifying tumor tissues.

### 3.2 Association between the expression of CX3CL1 and pan-cancer clinical characteristics

**FIGURE 2:**
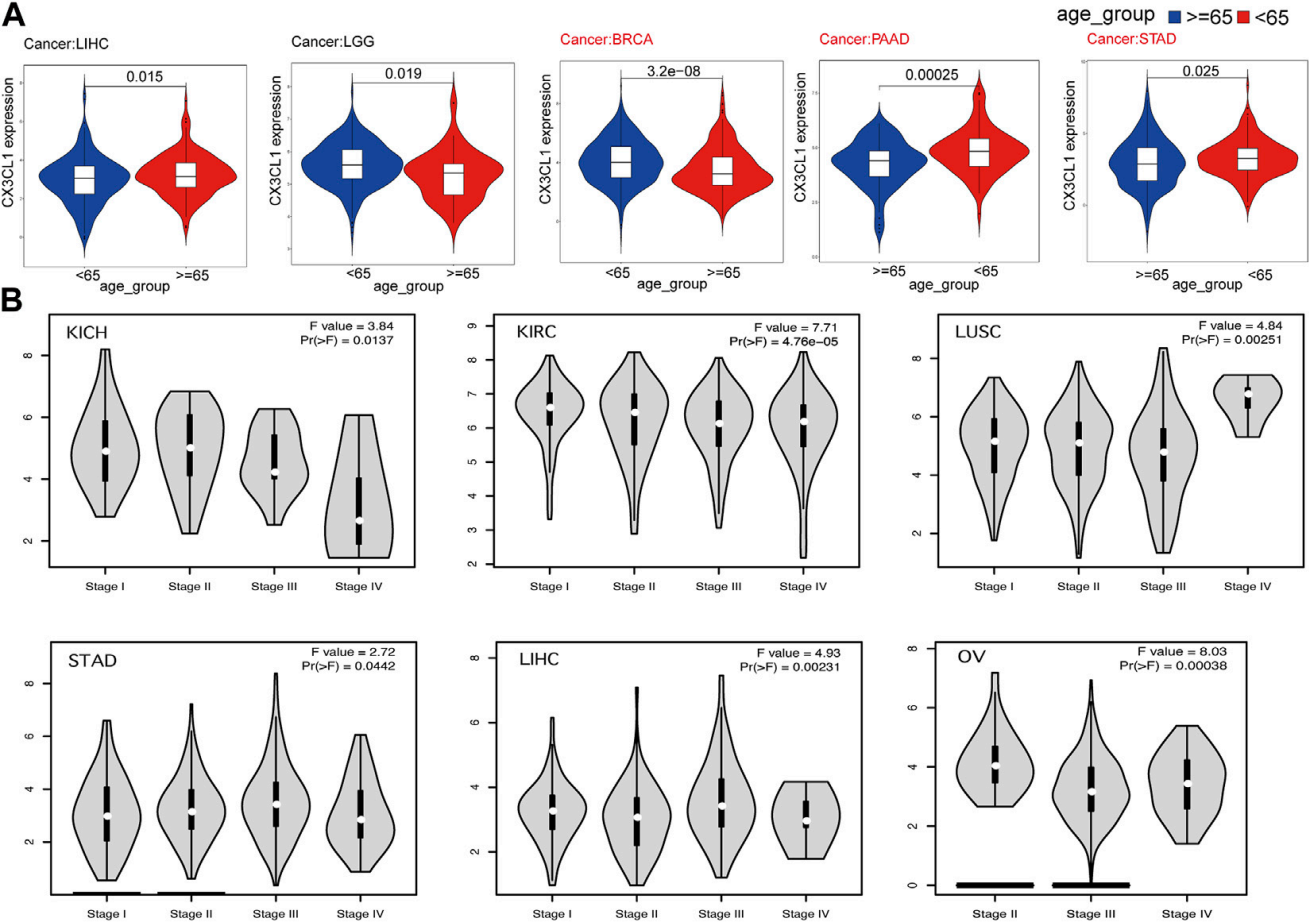
Correlations between the expression of CX3CL1 and clinicopathologic characteristics in pan-cancer. **(A)** CX3CL1 expression in patients of varying ages (<65 vs. ≥ 65 years) with different types of tumors, including LIHC, LGG, BRCA, PAAD, and STAD. **(B)** Correlations between differential expression of CX3CL1 and the pathological stages of KICH, KIRC, LUSC, STAD, LIHC, and OV.

Next, I set out to establish the clinical correlations of CX3CL1 expression. The disparities in CX3CL1 expression were discovered when patients suffering from various types of cancer were segmented into two categories, based on their ages. CX3CL1 expression was dramatically increased in older patients (≥65 years) with liver hepatocellular carcinoma (LIHC), while dramatically decreased in older patients with brain lower grade glioma (LGG), BRCA, PAAD, and STAD (Figure 2A). BRCA, PAAD, and STAD are members of the upregulated group, suggesting that the upregulated of CX3CL1 may be more related to the patients’ physical function. Other cancer patients showed no discernible relationship between age and CX3CL1 expression.

Further research was conducted to evaluate the relationship between CX3CL1 and tumor stages (Figure 2B). The data indicated that CX3CL1, across a variety of cancer types, was substantially associated with distinct pathological stages, including KICH, KIRC, ovarian serous cystadenocarcinoma (OV), LUSC, STAD, and LIHC. These findings indicate that CX3CL1 is related to the malignancy of cancer.

### 3.3 Analyses of CX3CL1 methylation levels and genetic alterations in pan-cancer

**FIGURE 3:**
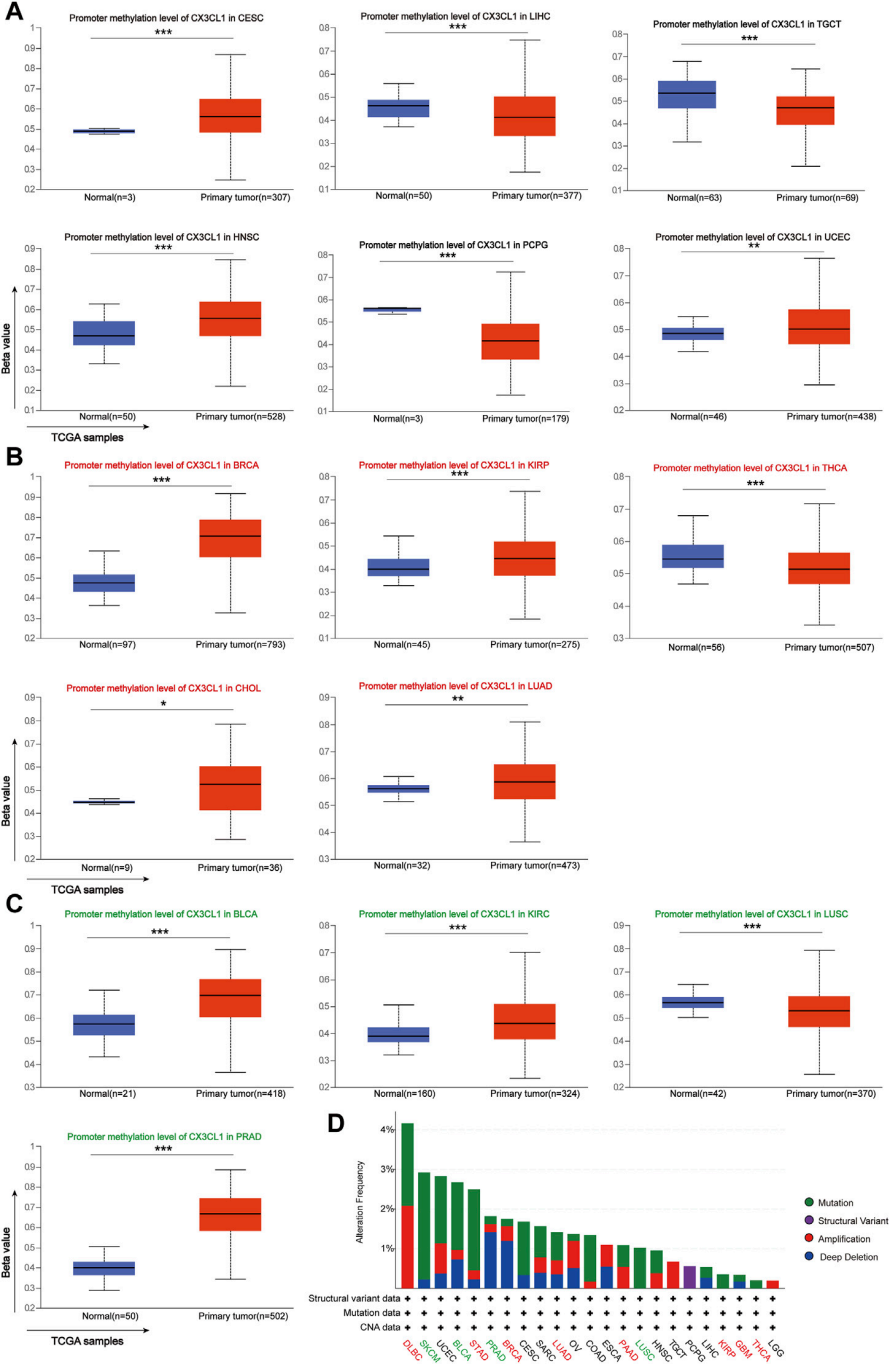
Analyses of pan-cancer genetic alterations and CX3CL1 methylation levels. **(A–C)** CX3CL1 methylation levels in different types of tumors **(A)** Unrelated group: CESC, LIHC, TGCT, HNSC, PCPG, and UCEC **(B)** Upregulated group: BRCA, KIRP, THCA, CHOL, and LUAD **(C)** Downregulated group: BLCA, KIRC, LUSC, and PRAD **(D)** The cBioPortal database showed the frequency of CX3CL1 mutations for various mutation types in upregulated group (red), downregulated group (green), and unrelated group (black). *p < 0.05; **p < 0.01; ***p < 0.001.

Previous research has shown that DNA methylation alters the expression of certain cancer driver genes, contributing to the development of cancer (Cui et al., 2022b). As recorded by the UALCAN database, there are 15 cancer types linked to CX3CL1 methylation. There were no significant differences between the expression of CX3CL1 in cancer samples and normal samples for six types of cancer: CESC, LIHC, TGCT, HNSC, PCPG, and UCEC (Figure 3A). In the other nine types of cancer, BRCA, KIRP, THCA, CHOL, LUAD, BLCA, KIRC, LUSC, and PRAD, there were significant differences between the expression of CX3CL1 in cancer samples and normal samples. Five cancer types belonged to the upregulated group (Figure 3B), while four cancer types belonged to the downregulated group (Figure 3C). Other types of cancer tissues and comparable normal tissues showed no discernible differences in CX3CL1 methylation. Moreover, the gene mutation profiles of specific patients may also influence gene expression patterns. Therefore, the cBioPortal database was used to conduct the investigation of CX3CL1’s genetic changes in various cancer types (Figure 3D). CX3CL1 gene mutations were found in 23 of 33 TCGA cancer types, including 8 types in the upregulated group and 4 types in the downregulated group. CX3CL1 is more likely to be mutated in cancers in which it is significantly upregulated. The result also showed that the mutation frequency of DLBC (4.17%), SKCM (2.93%), and UCEC (2.88%) ranked first, second, and third, respectively. Of note, missense mutations constitute the majority of CX3CL1 gene alterations. These results suggest a possible mechanism through which CX3CL1 may influence the incidence and development of cancer and offer explanations for its varying expression in tumors.

### 3.4 CX3CL1 prognostic analysis through all TCGA cancers

**FIGURE 4:**
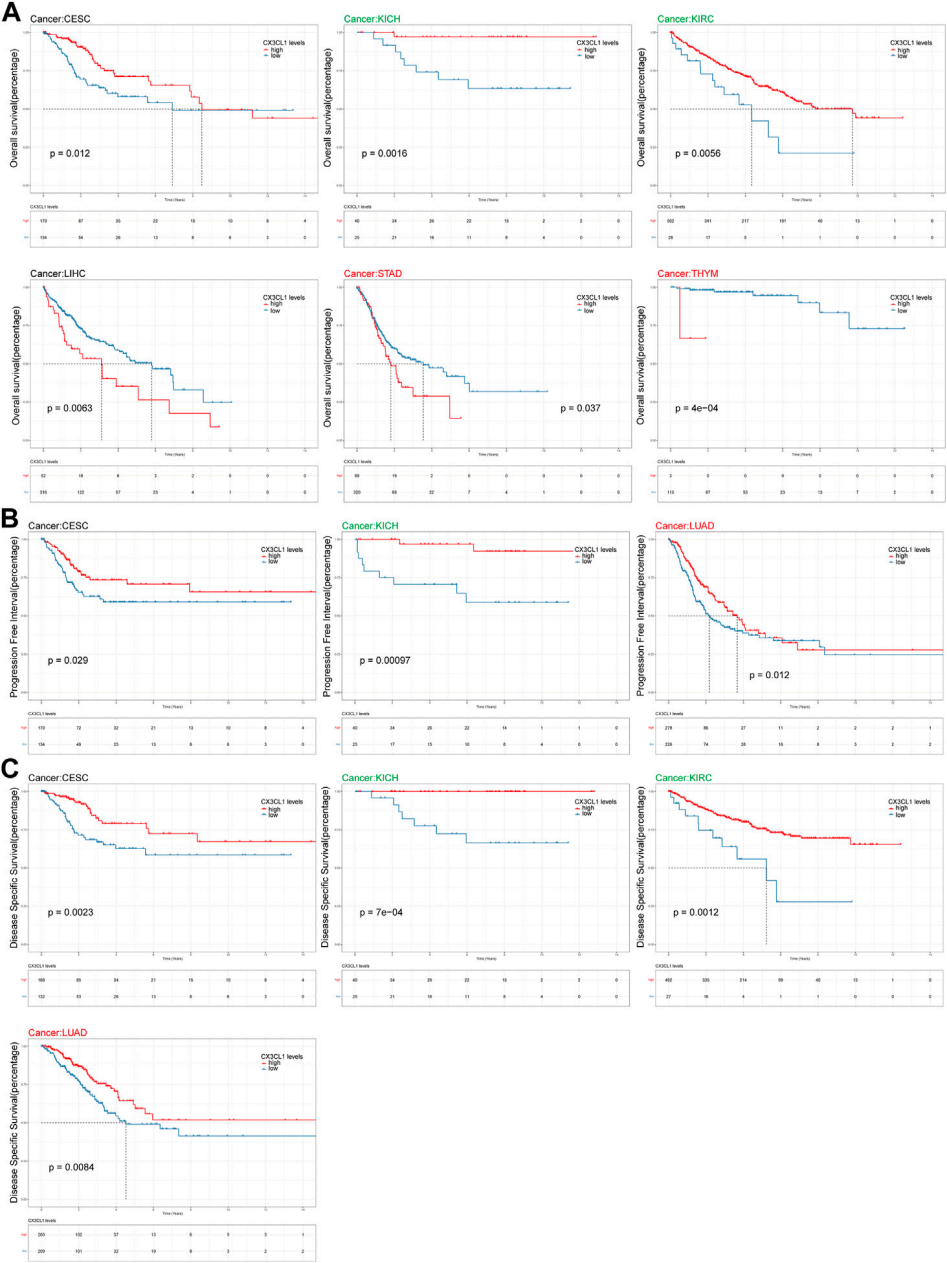
Analysis of link between CX3CL1 expression level and prognosis. **(A)** The Kaplan-Meier curve of overall survival (OS) in CESC, KICH, KIRC, LIHC, STAD, and THYM. STAD and THYM are members of the upregulated group, while KICH and KIRC are members of the downregulated group. **(B)** The Kaplan-Meier curve of progression free interval (PFI) in CESC, KICH, and LUAD. LUAD are members of the upregulated group, while KICH are members of the downregulated group. **(C)** The Kaplan-Meier curve of disease specific survival (DSS) in CESC, KICH, KIRC, and LUAD. LUAD are members of the upregulated group, while KICH and KIRC are members of the downregulated group.

Survival analysis were carried out to investigate the connection between CX3CL1 and the patient’s prognosis. Patients with elevated levels of CX3CL1 expression had a more favorable outcome in CESC, KICH, and KIRC, while high levels of CX3CL1 expression were substantially related with reduced OS in LIHC, STAD, and THYM (Figure 4A). Regarding the findings of the PFI analysis, there was a correlation between increased CX3CL1 expression and a considerably improved prognosis in CESC, KICH, and LUAD, which was partially compatible with the OS results (Figure 4B). Additionally, patients with higher CX3CL1 expression exhibited a higher DSS in CESC, KICH, KIRC, and LUAD (Figure 4C). These findings indicate that CX3CL1 is strongly related with the prognoses of cancer patients.

### 3.5 Immune cells infiltration of CX3CL1 in pan-cancer

CX3CL1 is known to induce its adhesive and migratory functions (Imai et al., 1997). In addition, T cells and monocytes are particularly drawn to the soluble form of CX3CL1, which is a powerful chemoattractant. Whereas the membrane-anchored form induces robust leukocyte adhesion to activated endothelial cells, indicating its essential role in regulation infiltrating immune cells (Bazan et al., 1997; Hasan et al., 2021).

**FIGURE 5:**
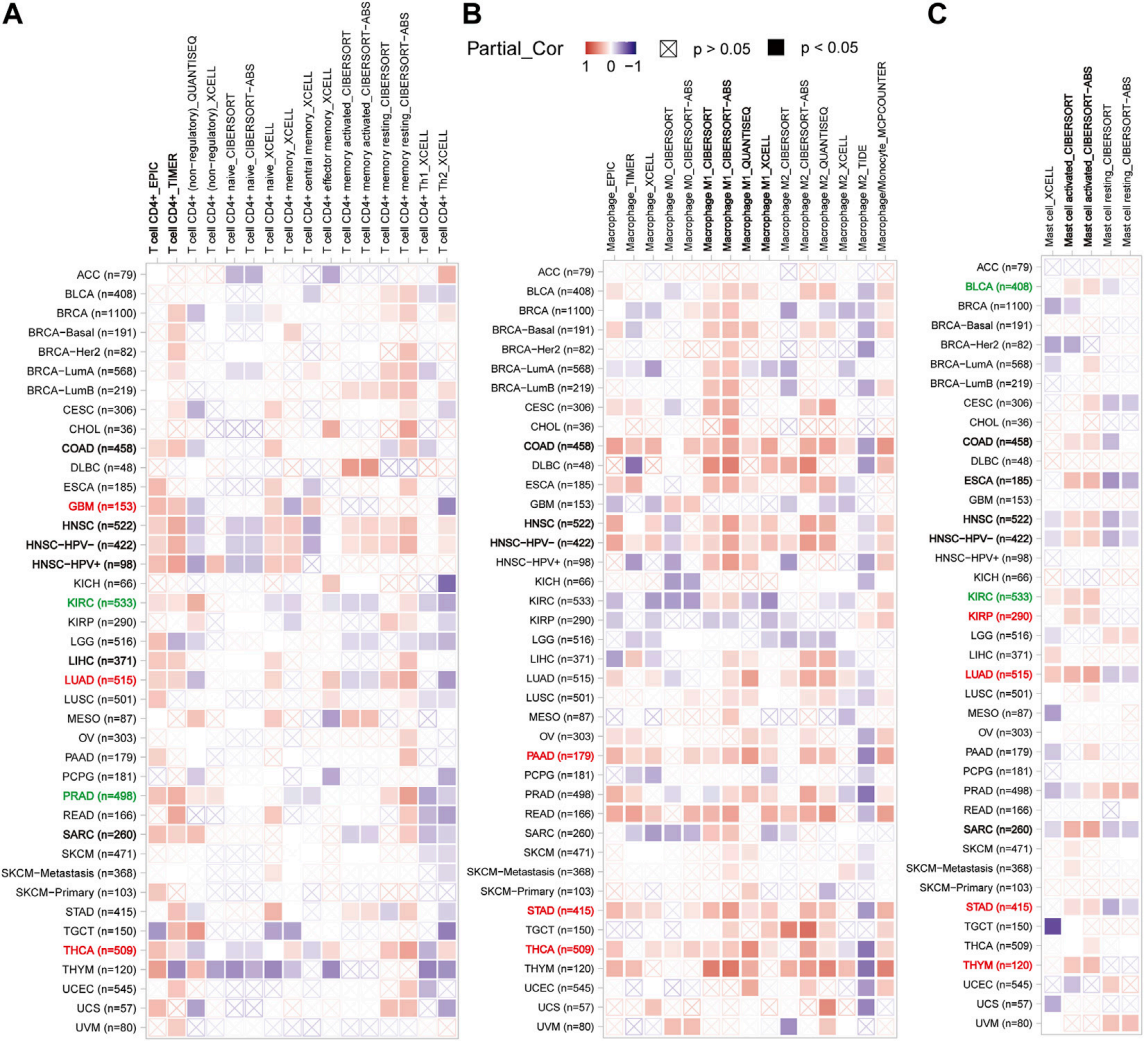
Correlation between CX3CL1 expression and the infiltration level of immune cells in pan-cancer (TIMER2). **(A)** The correlation heatmap depicted the correlation between CX3CL1 gene expression and the infiltration levels of CD4^+^ T cells in distinct cancer types utilizing EPIC, TIMER, QUANTISEQ, XCELL, CIBERSORT, and CIBERSORT-ABS methods. **(B)** The correlation heatmap depicted the correlation between CX3CL1 gene expression and the infiltration levels of macrophage in distinct cancer types utilizing EPIC, TIMER, XCELL, CIBERSORT, CIBERSORT-ABS, QUANTISEQ, TIDE, and MCPCOUNTER methods. **(C)** The correlation heatmap depicted the correlation between CX3CL1 gene expression and the infiltration levels of mast cells in distinct cancer types utilizing XCELL, CIBERSORT, and CIBERSORT-ABS methods.

In light of this, the TIMER2 database was utilized to investigate the possibility of a correlation between CX3CL1 and CD4^+^ T cell infiltration were observed in colon adenocarcinoma (COAD), LIHC, GBM, PRAD, KIRC, LUAD, HNSC, THCA, and sarcoma (SARC) based on the EPIC and TIMER algorithms (Figure 5A). GBM, LUAD, THCA are members of the upregulated group, while KIRC and PRAD are members of the downregulated group. Supplementary Figure S1 depicted the particular correlation scatter grams of the aforementioned tumor types. In addition, CX3CL1 expression demonstrated a considerable positive correlation with M1 macrophage cells in COAD, HNSC, PAAD, STAD, and THCA (Figure 5B). PAAD, STAD, and THCA are members of the upregulated group, which indicated CX3CL1 is more likely demonstrated a considerable positive correlation with M1 macrophage cells infiltration in which it is significantly upregulated. CX3CL1 also expression demonstrated a considerable positive correlation with activated mast cells (BLCA, COAD, ESCA, HNSC, KIRC, KIRP, LUAD, SARC, STAD, and THYM) infiltration in a variety of tumor types (Figure 5C). KIRP, LUAD, STAD, and THYM are members of the upregulated group, while BLCA and KIRC are members of the downregulated group. These findings highlight the critical function of CX3CL1 in regulating the tumor immune microenvironment.

### 3.6 Functional annotations of genes associated with CX3CL1

**FIGURE 6:**
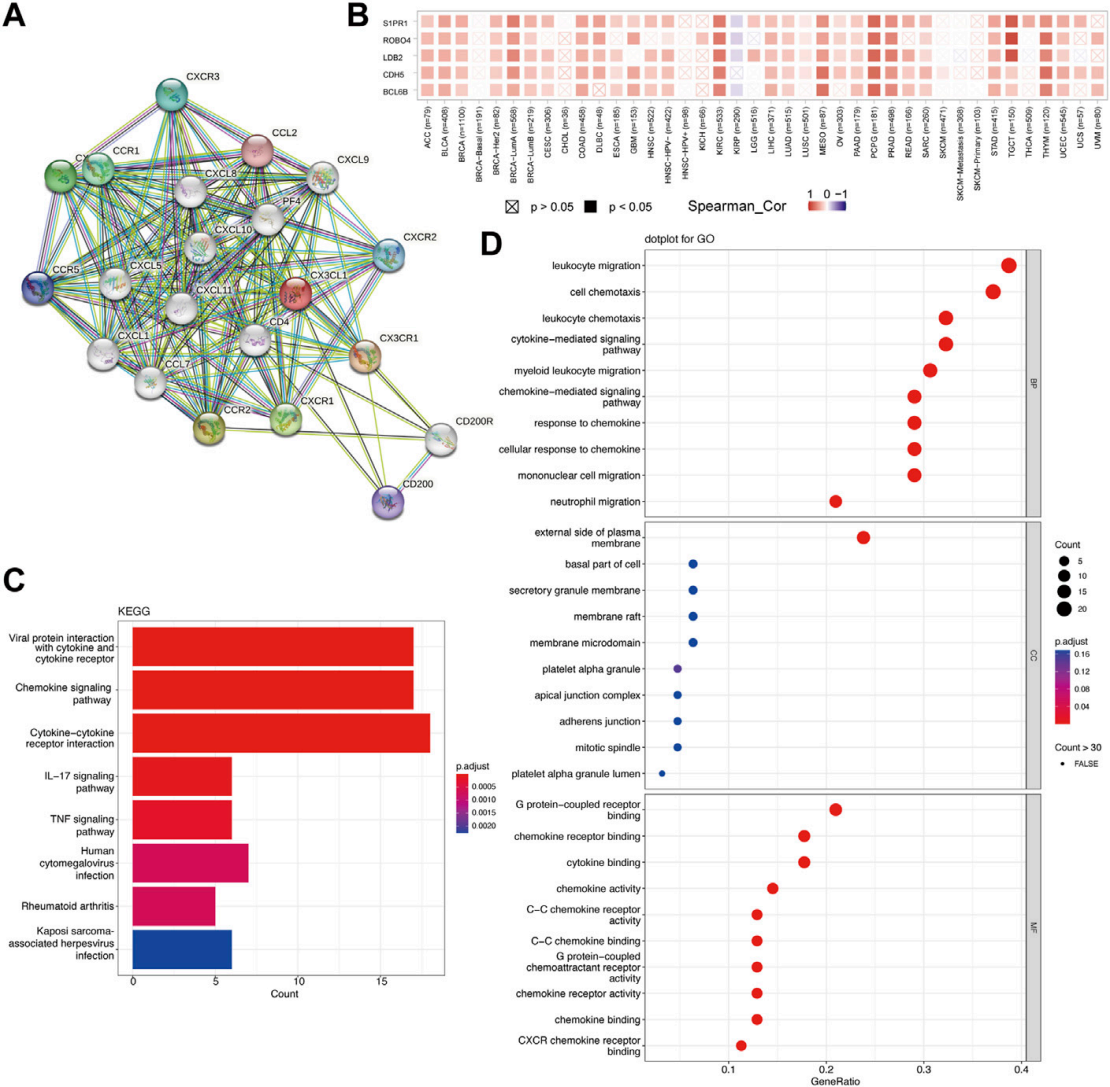
Functional enrichment analysis of CX3CL1 related genes. **(A)** Protein-protein interaction (PPI) network of 20 experimentally determined CX3CL1-interacting genes obtained from the STRING database. **(B)** The correlation heatmap showing the association between CX3CL1 expression level and CDH5, ROBO4, LDB2, BCL6B, and S1PR1 in various cancer types. **(C)** The bar plot of the Kyoto Encyclopedia of Genes and Genomes (KEGG)-enriched terms based on CX3CL1-interacting and CX3CL1-related genes. The column colors showed the significance of the adjusted *p*-value. **(D)** The dot bubble of gene ontology (GO)-enriched terms based on the two gene datasets, including biological process (BP), molecular function (MF), and cellular component (CC). The color of the dot indicated the significance of the adjusted *p*-value.

To study the probable molecular processes underlying carcinogenesis involving CX3CL1, STRING and GEPIA2 databases were employed to identify CX3CL1-interaction genes and CX3CL1-correlated genes for functional enrichment analyses, respectively. The PPI network in Figure 6A showed 20 experimentally discovered CX3CL1-interacting genes. Subsequently, following a search of the GEPIA2 database, the top 48 genes shown to have a correlation with CX3CL1 were identified (Cadherin 5 [CDH5] with R = 0.46, Roundabout Guidance Receptor 4 [ROBO4] with R = 0.44, LIM Domain Binding 2 [LDB2] with R = 0.44, BCL6B Transcription Repressor [BCL6B] with R = 0.43, and Sphingosine-1-Phosphate Receptor 1 [S1PR1] with R = 0.43) (Figure 6B). The correlation heatmap was created using maximum positive correlation coefficients, showing a strong connection between CX3CL1 and the five previously mentioned genes in the vast majority of TCGA tumors. Functional enrichments were then conducted on the two different groups of gene datasets described above. The KEGG analysis revealed probable pathways associated with CX3CL1, including “Viral protein interaction with cytokine and cytokine receptor”, “Chemokine signaling pathway”, and “Cytokine-cytokine receptor interaction” (Figure 6C). Furthermore, the most heavily enriched GO terms in the top 10 positions were presented, including “leukocyte migration,” “cell chemotaxis,” “leukocyte chemotaxis,” “cytokine-mediated signaling pathway,” and “myeloid leukocyte migration” for biological process, “external side of plasma membrane” for cellular component, and “G protein-coupled receptor binding,” “chemokine receptor binding,” and “cytokine binding” for MF, respectively (Figure 6D).

## 4 Discussion

In recent studies, CX3CL1 has been demonstrated to mediate immune cell survival and has lately been shown to be associated with the maintenance of cytotoxic T cell memory populations (Corr et al., 2014; Bottcher et al., 2015; Conroy et al., 2018). CX3CL1’s unique receptor CX3CR1 is expressed by a wide variety of immune cells and tumor cells in malignancies such as multiple myeloma, metastatic pancreatic cancer, prostate cancer and lymphocytic leukemia (Nishimura et al., 2002; Nakayama et al., 2010; Corcione et al., 2012; Huang et al., 2012; Wada et al., 2015; Liu et al., 2018). The expression profile analysis from the TCGA project, five of the eleven tumors (CHOL, KIRC, KIRP, THCA, and HNSC) with differential expression of CX3CL1, which had extremely high expression levels, whereas the remaining six cancer types had low CX3CL1 expression (BLCA, BRCA, KICH, LUAD, LUSC, and PRAD). However, when incorporating the normal samples from the GTEx project and expanding the sample size with the setting “Match TCGA normal and GTEx data” in the GEPIA2 database, only KICH still had low CX3CL1 expression, which was consistent with the prior result. What’s more, The GEPIA2 database uncovered eight novel cancer types (BRCA, DLBC, GBM, LUAD, PAAD, READ, STAD, and THYM) with high CX3CL1 expression, although BRCA had lower CX3CL1 expression previously. In addition, low expression of CX3CL1 was also observed in three other malignancies (ACC, KIRC, and SKCM). As indicated by the ROC analyses, CX3CL1 was able to identify tumor tissues from normal tissues in these cancers with significant changes in CX3CL1 expression. Of note, Figures 1A, B displayed divergent results, which may be attributable to the application of different algorithms and sample sizes with distinct databases. According to previous research, CX3CL1 expression was considerably elevated in THCA (Han et al., 2020), DLBC (Ferretti et al., 2011), BRCA (Park et al., 2012), STAD (Lv et al., 2014), PAAD (Xu et al., 2012), PRAD (Liu et al., 2018), LUAD (Liu et al., 2021), and GBM (Korbecki et al., 2020), while decreased in LUAD (Liu et al., 2019b), LUSC (Liu et al., 2019b), KIRC (Tsaur et al., 2011), and KICH (Tsaur et al., 2011). Therefore, further experimental and clinical validation of CX3CL1 expression in the other aforementioned cancers is still required.

Turning to the clinical significance of CX3CL1, I analyzed the connection between CX3CL1 expression and age as a starting point. In contrast to older patients with LGG, BRCA, PAAD, and STAD, CX3CL1 expression was considerably increased in those with LIHC. These findings suggested that aging alters the chemotaxis of immune cells in individuals with various malignancies, resulting in either altered tumor promotion or suppression, which was consistent with previous findings (Gupta et al., 2018; Nagai et al., 2021). Next, I attempted to determine the association between the CX3CL1 expression and certain pathological stages within various cancers. The expression of CX3CL1 was higher in LUSC, STAD and LIHC, and lower in KICH, KIRC, and OV during tumors progression, indicating that CX3CL1 plays a critical role in the carcinogenesis and progression of these cancers. As determined by pan-cancer prognostic analyses, high expression of CX3CL1 was substantially associated with improved OS, DSS, and PFI in CESC and KICH, OS and DSS in KIRC, and DSS and PFI in LUAD. In contrast, LIHC, STAD, and THYM patients with high CX3CL1 expression were significantly associated with worse OS. Of note, consistent with the result of the association between CX3CL1 and pathological stages in Figure 2, higher expression of CX3CL1 in STAD and LIHC patients was related with worse prognosis, whereas higher expression of CX3CL1 in KICH and KIRC patients was associated with better prognosis. Overexpression of CX3CL1 is typically associated with improved prognosis because of its ability to modulate the body’s immune response. Liu et al. discovered that CX3CL1 might exert an anti-tumorigenic effect on the KIRC cells line, hence contributing to the clinical benefit (Liu et al., 2022). Tsaur and his colleagues demonstrated that renal cells carcinoma of the chromophobe subtype had considerably reduced CX3CL1 gene expression relative to normal tissue, and that CX3CR1 and C-reactive protein were positively correlated (Tsaur et al., 2011). In addition to its favorable involvement in gastric adenocarcinoma regarding disease-free survival, CX3CL1 has also been documented to boost the immune system’s anti-tumor activity against LIHC and improve the prognosis of patients with hepatocellular carcinoma (Hyakudomi et al., 2008; Huang and Geng, 2010; Ashour et al., 2020; Yang et al., 2022).

In this study, I first evaluated the expression levels of CX3CL1 in human cancer and normal tissues across various tumors and revealed the difference of CX3CL1 methylation level between normal and tumor tissues, which partially explained the differential expression of CX3CL1 in various cancers. The preliminary data revealed a negative correlation between the level of methylation and the amount of mRNA expression for CX3CL1 in malignancies (Mi et al., 2022). Additionally, the CNA and mutation frequency of CX3CL1 in pan-cancer were investigated, and it was discovered that CX3CL1 undergoes considerable gene alterations in various cancer types, with the highest proportion of missense mutation and amplification. Previous study also showed that the chemotherapy-sensitive Hodgkin lymphoma patients had frequent gains of 16q13, a chromosomal region known to house genes that regulate T-cells trafficking or NF-ĸB activation (CX3CL1, CCL22, CCL17, DOK4, and IL10) (Slovak et al., 2011). Whether CX3CL1 functions as an oncogene and the precise mechanisms that regulate CX3CL1 remain to be determined.

There is growing evidence that the tumor microenvironment, including cancer-associated fibroblasts, tumor-associated immune cells, and the extracellular matrix, interacts with cancer cells to influence tumor development, metastasis, response to immunotherapy, and prognosis (Bindea et al., 2013; El-Kenawi et al., 2019; Ligorio et al., 2019; Zhang et al., 2021b). Consequently, the relationship between CX3CL1 expression and cancer immunity was investigated. CX3CL1 expression was found to be positively connected with CD4^+^ T cells infiltration across most TCGA cancer types, as well as activated mast cells and M1 macrophage cells across several particular tumor types, indicating that CX3CL1 may also reflect the immune status in various cancers. The role of CX3CL1 in anti-tumor immunity is the subject of several investigations that provide inconsistent results. According to a number of studies, CX3CL1 plays a critical role in fostering robust anti-tumor activity by recruiting NK cells and T cells into the tumor microenvironment (Xin et al., 2005; Zeng et al., 2005; Nukiwa et al., 2006; Siddiqui et al., 2016). Thus, more research is needed to fully understand the molecular mechanisms underpinning the correlation between CX3CL1 expression and other immune cells infiltration.

KEGG and GO enrichment analyses, to define distinct pathways and biological processes in a variety of malignancies. The KEGG enrichment analysis revealed the association of CX3CL1 on tumorigenesis. The GO enrichment analysis shown the enrichment for several receptor bindings and chemokine activity processes. Together, these results raise the possibility that CX3CL1 is a therapeutic treatment target in a variety of malignancies and that the chemokine signaling pathway is involved in its functional mechanisms.

In conclusion, our study performed a systematic, pan-cancer analysis of CX3CL1, assessing the potential association between CX3CL1 expression and clinical outcomes, pathological stages, and immune cell infiltration in a wide range of cancer types. This will help paint a clearer picture of the roles CX3CL1 plays in human cancers.

## Data Availability

The original contributions presented in the study are included in the article/Supplementary Material, further inquiries can be directed to the corresponding author.
