# Supplementary material for: A systematic pan-cancer analysis reveals the clinical prognosis and immunotherapy value of C-X3-C motif ligand 1 (CX3CL1)
Source: Front Genet. 2023 Apr 20;14:1183795. doi: 10.3389/fgene.2023.1183795 (PMC10157490; doi:10.3389/fgene.2023.1183795)
Supplement: Supplementary file 4 [file Table2.DOCX]

Supplementary Material

A Systematic Pan-Cancer Analysis Reveals the Clinical Prognosis and Immunotherapy of C-X3-C Motif Ligand 1 (CX3CL1)

Yidi Sun*

*** Correspondence:** Yidi Sun: 20086000210038@hainanu.edu.cn

# Supplementary Figures and Tables

## Supplementary Figures


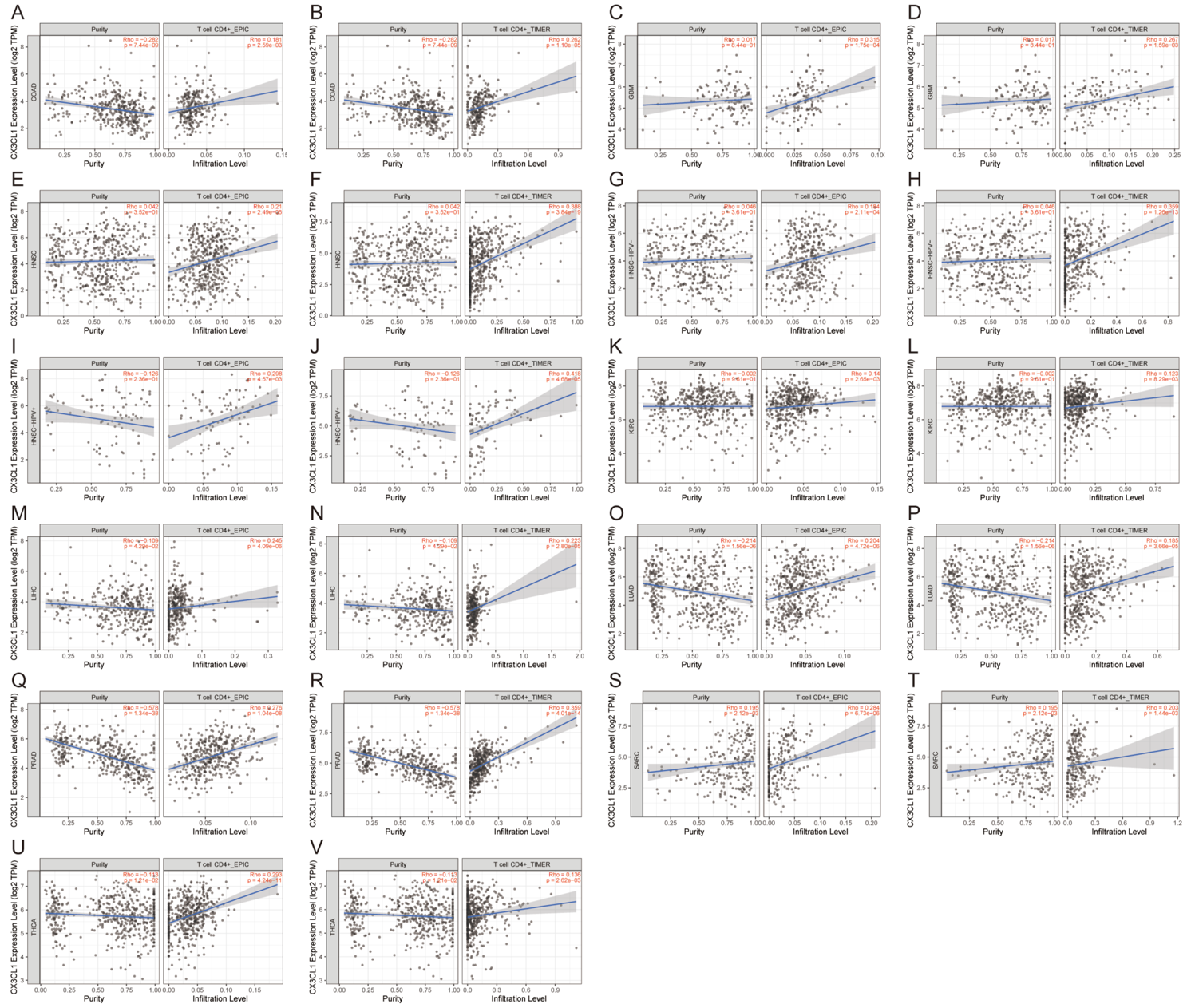


Supplementary Figure 1. The correlation scatter grams between CX3CL1 expression and the infiltration level of CD4^+^ T cells in nine cancer types. (A-V) The scatter plots depicting the association between CX3CL1 gene expression and CD4^+^ T cells infiltration estimation value using the EPIC and TIMER methods, in (A-B) COAD, (C-D) GBM, (E-F) HNSC, (G-H) HNSC-HPV-, (I-J) HNSC-HPV+, (K-L) KIRC, (M-N) LIHC, (O-P) LUAD, (Q-R) PRAD, (S-T) SARC, (U-V) THCA.

Supplementary Table 1. 48 genes positively linked with CX3CL1 with a Pearson product-moment correlation coefficient (PCC) greater than 0.35 were selected from the GEPIA2 database.

| Gene Symbol | Gene ID | PCC |
| --- | --- | --- |
| CDH5  ROBO4  LDB2  BCL6B  S1PR1  CRIM1  RAPGEF5  EDN1  GPR4  EPAS1  ECSCR  TIE1  GIMAP8  CASKIN2  NEDD9  SHROOM4  VWF  ADGRL4  DOCK4  ESAM  DLL4  AKAP2  MAML2  TEK  GIMAP5  ARHGAP29  PECAM1  CD93  CLEC14A  CXorf36  MMRN2  EMCN  NOTCH4  RBMS2  RP11-389C8.2  RP11-134L10.1  TNIP1  ARID5A  ARHGEF15  RGL1  HECW2  NRP1  LRRC32  CALCRL  PDGFB  ZNF366  RP11-693N9.2  SUSD6 | ENSG00000179776.17  ENSG00000154133.14  ENSG00000169744.12  ENSG00000161940.10  ENSG00000170989.8  ENSG00000150938.9  ENSG00000136237.18  ENSG00000078401.6  ENSG00000177464.4  ENSG00000116016.13  ENSG00000249751.3  ENSG00000066056.13  ENSG00000171115.3  ENSG00000177303.9  ENSG00000111859.16  ENSG00000158352.15  ENSG00000110799.13  ENSG00000162618.12  ENSG00000128512.19  ENSG00000149564.11  ENSG00000128917.6  ENSG00000241978.9  ENSG00000184384.13  ENSG00000120156.20  ENSG00000196329.10  ENSG00000137962.12  ENSG00000261371.5  ENSG00000125810.9  ENSG00000176435.6  ENSG00000147113.16  ENSG00000173269.13  ENSG00000164035.9  ENSG00000204301.6  ENSG00000076067.11  ENSG00000261269.1  ENSG00000272941.1  ENSG00000145901.14  ENSG00000196843.15  ENSG00000198844.10  ENSG00000143344.15  ENSG00000138411.10  ENSG00000099250.17  ENSG00000137507.11  ENSG00000064989.12  ENSG00000100311.16  ENSG00000178175.11  ENSG00000235505.7  ENSG00000100647.7 | 0.46  0.44  0.44  0.43  0.43  0.42  0.42  0.41  0.41  0.41  0.41  0.4  0.4  0.4  0.39  0.39  0.39  0.39  0.39  0.39  0.38  0.38  0.38  0.38  0.38  0.38  0.38  0.38  0.38  0.37  0.37  0.37  0.37  0.37  0.37  0.37  0.37  0.37  0.37  0.37  0.37  0.36  0.36  0.36  0.36  0.36  0.36  0.36 |
